# Food Away from Home Frequency, Diet Quality, and Health: Cross-Sectional Analysis of NHANES Data 2011–2018

**DOI:** 10.3390/nu14163386

**Published:** 2022-08-18

**Authors:** Sayaka Nagao-Sato, Marla Reicks

**Affiliations:** Department of Food Science and Nutrition, University of Minnesota, St. Paul, MN 55108, USA

**Keywords:** NHANES, HEI-2015, meals prepared away from home, diet quality, health

## Abstract

The consumption of meals prepared away from home (FAFH) in the U.S. has been commonly linked to overall poorer diet quality; however, less is known about the relationship with health parameters. The aim of this cross-sectional study was to assess the diet quality and health parameters of adults who reported the frequency of obtaining or eating FAFH using a combination of four 2-year cycles of National Health and Nutrition Examination Survey (NHANES, 2011–2018) data. Data from 14,999 adults aged 20 to 65 years were used to examine the associations between diet and health outcomes and the weekly frequency of FAFH. Multivariable regression models were used to compare Healthy Eating Index-2015 (HEI-2015) scores by FAFH frequency, adjusting for sociodemographic variables. Adults with more frequent FAFH meals were more likely to be younger, male, lower-income, and unmarried. Total HEI-2015 scores and component scores for greens and beans, total and whole fruits, whole grains, saturated fats, and added sugars were significantly lower in those consuming >2 FAFH meals/week vs. ≤2 FAFH meals/week. BMI and waist circumference tended to be higher for women having more frequent FAFH meals. More frequent FAFH meals among adults were associated with poorer diet quality and weight outcomes, building on results from earlier studies.

## 1. Introduction

The consumption of foods/meals prepared away from home (FAFH) in the U.S. has generally increased since 1977 [1]. National Health and Nutrition Examination Survey (NHANES) data from 2013–2014 showed that 34% and 16% of daily energy for U.S. adults was provided by FAFH and fast food, respectively [2]. Studies have shown associations between intake of FAFH and poorer diet quality [2,3,4,5,6,7,8]. Fast-food and full-service restaurant use among adults was associated with increased daily intake of energy, total fat, saturated fat, cholesterol, and sodium based on NHANES data from 2003–2010 [3]. Another study showed that higher FAFH consumption frequency was associated with lower levels of fruit and vegetable intake and higher body mass index [4]. National U.S. surveys in 2005–2008 found that FAFH, compared to food at home (based on where the food was obtained), was higher in saturated fat, sodium, and cholesterol and lower in dietary fiber among adults [5]. Conrad et al. [6] reported higher total Healthy Eating Index-2015 (HEI-2015) scores for foods at home (61.8) compared to FAFH (54.5) based on NHANES 2005–2016 among adult participants. Consumption of FAFH from fast-food outlets was associated with lower odds of meeting Dietary Approaches to Stop Hypertension dietary patterns [7] and higher intakes of energy and empty calories and lower intakes of vegetables, whole grains, and fiber [8]. In addition to negative effects on diet quality, FAFH frequency has been associated with higher BMI and obesity [4,7,9], greater risk of type 2 diabetes [10], and lower serum concentrations of high-density lipoprotein (HDL) cholesterol [9].

FAFH consumption frequency has been explained by several individual and environmental considerations, including the amount of time spent cooking at home [11], income [12], and the number and variety of restaurants available [2]. The mean per capita time spent cooking based on U.S. time use data from 2016 also varied among individuals and environmental factors [13]. For example, women spent 50 min/d cooking vs. 20 min/d for men, middle-aged to older adults spent 38 min/d cooking vs. 24 min/d for young adults, and those not in the labor force spent 55 min/d cooking vs. 26 min/d for those working full-time. Data from the National Household Acquisition and Purchase Survey [14] showed that the frequency of FAFH acquisition was higher among younger adults, with a peak at 35–44 years [2]. This finding was attributed to a higher extent of labor force participation, more active social networks, and a greater desire for convenience among younger vs. older adults. Race and sex were not significant predictors of FAFH acquisition, whereas age was the primary driving factor. A positive correlation was observed between the frequency of FAFH acquisition and income in all age groups [2], consistent with the potentially higher cost of FAFH compared to home-prepared meals.

This study updated previous research using NHANES data from 2005–2010 to identify relationships between FAFH, dietary intake, and metabolic biomarkers of disease [9]. In addition, this study examined relationships among FAFH, diet quality assessed with HEI-2015 scores, and demographic characteristics, including age, sex, and income. Inverse associations were observed between diet quality, as measured with HEI-2015 scores, and risk of mortality from all causes, cardiovascular disease, and cancer in a multiethnic cohort [15]. The aim of this study was to examine associations among FAFH frequency, dietary quality, metabolic biomarkers of disease, and selected demographic factors of adults using NHANES (2011–2018) data.

## 2. Materials and Methods

### 2.1. Study Design and Participants

NHANES is a nationally representative survey that includes an assessment of dietary intake and behaviors conducted in 2-year cycles by the U.S. National Center for Health Statistics, Centers for Disease and Control and Prevention (CDC) [16]. The survey was conducted with a combination of in-home interviews and physical measures at a mobile examination center (MEC). Details of the survey are available at the CDC website [17]. Cross-sectional data for the current study included data from adults aged 20 to 65 years who participated in a combination of 4 NHANES cycles (2011–2018) (*n* = 17,548). NHANES cycles are designed to be combined to increase sample size and produce estimates with greater precision and smaller sampling error. Data from a total of 14,999 participants were used for this study after excluding those with non-reliable one-day dietary recall records (*n* = 2156), those who identified as pregnant (*n* = 247), and those who were breastfeeding (*n* = 146). The National Center for Health Statistics Institutional Review Board for the Ethics Review Board approved NHANES data collection [18]. Written informed consent was obtained from participants before data collection. The University of Minnesota Institutional Review Board determined that analysis of deidentified, publicly available NHANES data was not research involving human subjects as defined by U.S. Department of Health and Human Services and Food and Drug Administration regulations.

Demographic characteristics reported in the current study included age, sex (male and female), race/ethnicity (Mexican/other Hispanic, non-Hispanic White, non-Hispanic Black, non-Hispanic Asian, and other/multi-racial), marital status (married, living with partner and not married), country of birth (born in the U.S. and not born in the U.S.), and education level (less than high school, high school/GED, some college, and college graduate or above). The family income-to-poverty ratio (PIR) was dichotomized as <1.3 and ≥1.3. Families under 1.3 PIR are eligible to receive Supplemental Nutrition Assistance Program (SNAP) benefits in the U.S. [19]. All variables were used in the regression analyses as covariates.

### 2.2. Meals Prepared Away from Home and Dietary Intake

In the MEC, as part of the Diet Behavior and Nutrition questionnaire [20], trained staff asked participants to report the number of meals that were prepared away from home during the past seven days in places such as restaurants, fast-food places, food stands, grocery stores, or vending machines. Two FAFH frequency categories were based on the median frequency of meals prepared away from home (≤2 meals/week and >2 meals/week).

Dietary intake data were obtained from the first day’s 24 h dietary recall interview conducted in person by trained interviewers in the MEC using the Automated Multiple-Pass Method (AMPM) developed by the U.S. Department of Agriculture (USDA) [21]. The reliability of the dietary recall records was evaluated based on the completion of the first four of the five AMPM steps and the identification of food/beverages consumed for each reported eating occasion. In-person dietary recall data from day 1 only were used rather than an average of day 1 and day 2 recalls collected by telephone to prevent a reduction in sample size and study power and to avoid using dietary intake data collected using different methods. Food group intake data were taken from the Food Patterns Equivalents Database for 2011–2018, which converted the recorded amounts of foods and beverages consumed 24 h prior to the interviews into 37 USDA food pattern components based on the Food and Nutrient Database for Dietary Studies [22]. Diet quality was evaluated using HEI-2015 scores based on the USDA food patterns equivalents, which included nine adequacy and four moderation component scores, indicating alignment with the 2015–2020 Dietary Guidelines for Americans [23]. The adequacy component scores included total fruit, whole fruit, total vegetables, greens and beans, whole grains, dairy, total protein foods, seafood and plant proteins, and fatty acids, and the moderation component scores included refined grains, sodium, added sugars, and saturated fats. Higher scores of the nine HEI-2015 adequacy components reflected higher intakes of total fruits, whole fruits, total vegetables, greens and beans, whole grains, dairy, total protein foods, seafood and plant proteins, and fatty acids. Higher scores of the four moderation components indicated lower intakes of refined grains, sodium, added sugars, and saturated fats, which are food groups and nutrients for which limited consumption is encouraged. The total HEI-2015 score was the sum of all component scores for a possible 100 points, with higher scores being favorable.

### 2.3. Anthropometric Measures and Biomarkers of Chronic Disease

The anthropometric measurements used in this study included height, weight, and waist circumference using standard anthropometric measurement protocols [24]. Body mass index (BMI = weight in kg/height in m^2^) was calculated based on measured height and weight. 

Glycohemoglobin and lipid profile parameters were assessed based on laboratory methodology according to established procedures [25]. Glycohemoglobin, total cholesterol, HDL and low-density lipoprotein (LDL) cholesterol, and triglycerides were measured in the morning session in a subsample of fasted participants who had been fasting for 8 to <24 h. 

### 2.4. Data Analyses

SAS Survey software (version 9.4, Statistical Analysis System, Cary, NC, USA) was used to account for the multistage probability sampling design of NHANES in the statistical analyses. The SAS Surveyfreq and Surveylogistic procedures and Rao–Scott chi-square test were used to describe sociodemographic characteristics by FAFH frequency and identify associations. The SAS Surveyreg procedure was used for multivariable regression models to compare HEI-2015 scores, anthropometric measures, and biomarkers of chronic disease (dependent variables) by FAFH frequency (independent variables), adjusting for sociodemographic variables that were significantly associated with FAFH frequency. For anthropometric measures, the models were evaluated by sex separately. In the SAS procedures, appropriate strata, cluster, and weight statements were used to account for sampling bias and the survey design. The level of significance was set at *p* < 0.05 with Bonferroni–Holm correction applied to adjust the *p* value for multiple comparisons.

## 3. Results

Among 14,999 adults aged 20–65 years, 12 participants responded ‘don’t know’ regarding FAFH frequency and were excluded from the analysis. About half of the participants (*n* = 7362, 49%) reported having more than two FAFH meals a week. The frequency of adults who reported eating meals prepared away from home per week was 0 meals (*n* = 2994), 1 meal (*n* = 2312), 2 meals (*n* = 2319), 3 meals (*n* = 1928), 4 meals (*n* = 1221), 5 meals (*n* = 1211), 6 meals (*n* = 422), 7 meals (*n* = 876), and > 7 meals (*n* = 1704).

Sociodemographic characteristics, including age, PIR, sex, race/ethnicity, education level, marital status, and country of birth, are presented in Table 1. Adults who had >2 FAFH meals/week were more likely to be younger. Using FAFH ≤ 2 meals/week as the reference category, significantly higher odds of >2 FAFH meals/week were observed among participants who were male and were not married. Significantly lower odds of >2 FAFH meals/week were observed for those who had a PIR < 1.3. Participants who were Mexican Americans, non-Hispanic Black or Asian had significantly lower odds of having >2 FAFH meals/week vs. Non-Hispanic White. Those with high school/GED, some college, and college degrees or above were more likely to have >2 FAFH meals/week compared to those with less education.

Table 2 shows differences in diet quality by FAFH frequency. The total HEI-2015 score was higher among participants in the ≤2 FAFH meals/week group compared to those in the >2 FAFH meals/week group (*p* < 0.0001). In addition, component scores for greens and beans, total fruits, whole fruits, whole grains, saturated fat, and added sugars were significantly higher for those consuming ≤2 FAFH meals/week vs. >2 meals/week (*p* = <0.0001 to 0.0009). Other adequacy and moderation component scores were not significantly different between FAFH frequency groups.

BMI and waist circumference tended to be higher for women consuming >2 FAFH meals/week vs. ≤2 meals/week but were not significantly different after correction for multiple comparisons (Table 3). Similarly, glycohemoglobin and triglyceride concentrations tended to be higher among participants consuming FAFH more often but were not significantly different after correction for multiple comparisons. Cholesterol concentrations did not significantly differ between the two FAFH frequency groups.

## 4. Discussion

Sociodemographic characteristics regarding age, sex, income, and education level were associated with FAFH frequency. Total HEI-2015 index scores for those having >2 FAFH meals/week were lower than those having ≤2 FAFH meals/week. BMI and waist circumference tended to be higher among women who had >2 vs. ≤2 FAFH meals/week. In general, the results regarding diet quality were consistent with previous studies [1,2,3,4,5,6,7,8,9], indicating that overall diet quality may be improved for some demographic subgroups by reducing the frequency of FAFH.

Participants in the >2 FAFH meals/week group were more likely to be younger, have higher household income, and attain a higher education level than those in the ≤2 FAFH meals/week group, which was consistent with previous reports by USDA-ERS [5] and Seguin et al. [4]. Saksena et al. suggested that younger adults may have active social networks, which would involve more frequent FAFH [2]. In addition, among university students, the perception of engaging in indulgent vs. sensible eating with friends increased feelings of interpersonal closeness [26], which might increase the frequency of FAFH with others among younger adults. The findings from the current study regarding positive associations among higher household income and higher levels of education and the FAFH frequency of foods/meals were similar to the findings of Saksena et al. [2]. Working full-time may result in higher income to facilitate a greater frequency of meals away from home but also possibly less time for preparing meals at home. Our results also showed that those in the >2 FAFH meals/week group were more likely to be male compared to the ≤2 FAFH meals/week group. A previous study similarly found that men reported consuming FAFH more often than women, which was associated with a higher BMI and lower daily fruit and vegetable intake [4]. One explanation for this relationship is the role of time spent at work, which was positively associated with FAFH in a U.S. national sample, with a greater influence on married men vs. married women [27]. 

Compared to those consuming ≤2 FAFH meals/week in the current study, the diet quality of those consuming >2 FAFH meals/week group was lower for total HEI scores and component scores for greens and beans, total fruits, whole fruits, whole grains, and added sugars. Consistent with these findings, a previous study reported lower HEI-2015 component scores for greens and beans, total fruits, whole grains, and added sugars in foods from restaurants, fast food, and mixed sources than from foods at home [28]. In addition, Liu et al. [29] observed higher diet quality (NHANES 2017–2018) regarding total fruits, whole fruits, and whole grains consumed from grocery stores compared to restaurants based on HEI-2015 scores. The greater availability of healthy foods in grocery stores than in full-service and fast-food restaurants supports the current findings, as measured by the Nutrition Environment Measures Survey in different regions of the U.S. [30,31]. Therefore, intakes of fruits and whole grains based on the food source could contribute to the higher HEI-2015 scores for those consuming ≤2 FAFH meals/week compared to those consuming >2 FAFH meals/week. Conversely, slightly higher HEI-2015 scores for added sugars were observed in foods consumed from restaurants than from grocery stores in a previous study (NHANES 2017–2018) [29]; however, in the current study, those consuming >2 FAFH meals/week had lower HEI-2015 component scores for added sugars than those consuming ≤2 FAFH meals/week. Restaurants may primarily serve meals where sugar-sweetened beverages (SSBs) account for added sugar content, while grocery stores stock a wide variety of foods high in added sugars in addition to SSBs.

Studies comparing diet quality by food source differ in methodology, which could contribute to conflicting results. In the current study, participants were divided into frequency groups by reported weekly frequencies of FAFH meals. Total HEI-2015 scores were higher among those consuming ≤2 FAFH meals/week compared to those consuming >2 FAFH meals/week. Another approach by Patetta et al. [28] involved the calculation of total HEI-2015 scores using diet recall information from NHANES 2011–2014 based on days when foods from specific food sources were consumed. Total HEI-2015 scores were 61.5 for the at-home group and 63.0 for the restaurant group, which were not generally consistent with the results in the current study. Other studies have compared the nutrient density of over- and under-consumed food components by food source based on NHANES data as a measure of diet quality rather than the HEI-2015 [1,9]. These studies showed that food at home had higher calcium (mg/100 kcal) and fiber densities (g/1000 kcal) and lower sodium density (mg/1000 kcal) compared to FAFH foods. In the current study, HEI-2015 component scores including foods high in fiber, such as greens and beans, total fruits, whole fruits, and whole grains, were higher in the ≤2 FAFH meals/week group compared to the >2 FAFH meals/week group, consistent with other studies [1,9]. However, the HEI-2015 component scores for sodium and dairy (a common food source of calcium) were not different between FAFH frequency groups in the current study.

BMI and waist circumference tended to be higher among women having >2 FAFH meals/week in the current study compared to those having ≤2 FAFH meals/week, consistent with BMI findings in previous studies [4,7,8,9]. The tendency for lower BMI and waist circumference in the current study among women having less frequent FAFH meals/week may be partially explained by their consumption of more greens and beans and fruits. The association between lower BMI and higher intakes of fruit and vegetables and legumes was reported in previous studies involving adults [32,33]. A previous study involving NHANES 2005–2010 data [9] did not show adverse relationships between serum concentrations of total and LDL cholesterol and the frequency of food away from home, consistent with the results of the current study.

Implications for public health based on the findings of the current study are that those who consume FAFH, even at a low weekly frequency, may have lower intakes of specific foods, such as greens and beans, total fruits, whole fruits, and whole grains, and greater intakes of added sugars, which could contribute to lower total diet quality scores compared to those who have ≤2 FAFH meals/week. Because higher scores indicate greater alignment between foods and beverages consumed and the Dietary Guidelines for Americans [23], a lower frequency of FAFH is recommended. Interventions to improve diet quality could be tailored by dietitians to address healthy eating patterns in different settings where individuals obtain and consume foods [34]. A complementary approach would be to promote the increased frequency of home cooking. Cooking dinner at home ≥7 times per week was associated with higher HEI-2015 scores than fewer times per week among U.S. adults (NHANES 2007–2010) [35]. Notably, total vegetable and empty calorie HEI-2010 scores based on intake during dinner or the whole day were higher (more favorable) for those who cooked dinner at home 6–7 times per week compared to fewer times per week (NHANES 2007–2010) [36].

This study used nationally representative data for 1 day to indicate FAFH frequency based on the food preparation source, including those sold at grocery stores, which could contribute to greater accuracy when considering differences in the diet quality of food at home vs. FAFH. However, several limitations are acknowledged. First, this study only considered meals that were prepared away from home based on the Diet and Nutrition Behavior Questionnaire without including snacks prepared away from home. Additionally, the HEI-2015 scores in this study may not have been based on a day when foods that were not prepared at home were consumed. However, the use of the Diet and Nutrition Behavior Questionnaire questions about the frequency and use of foods not home-prepared to group participants into FAFH frequency groups did not introduce misclassification bias, as was present in other studies based on purchase and consumption locations. Statistically significant differences in HEI scores were based on small numerical differences due to the large sample sizes. However, individual component scores can be examined collectively to reveal a pattern of diet quality, which can provide more information about the practical vs. statistical significance of the results. Lastly, dietary quality could also have been affected by the meal environment context, including eating in the presence or absence of others, which has been shown to influence the type of foods consumed in various settings [37] as well as food costs [6].

## Figures and Tables

**Table 1:** Descriptive assessment for regression models by FAFH frequency (*n* = 14,987).

	FAFH			
	≤2 Meals/Week (*n* = 7625)	>2 Meals/Week (*n* = 7362)	OR ^1^ (95%CI)	*p* ^2^
	----- LS means (SE) -----			
Age	44.8 (0.24)	40.7 (0.33)		<0.0001
	---------- *n* (%) ----------			
Sex				
Female	4279 (56.6)	3304 (44.0)	ref	
Male	3358 (43.4)	4058 (56.0)	1.66 (1.53, 1.81)	<0.0001
Race/ethnicity				
Non-Hispanic White	2425 (59.8)	2713 (66.0)	ref	
Mexican American	1247 (10.7)	936 (8.3)	0.70 (0.60, 0.81)	0.037
Other Hispanic	911 (7.6)	691 (6.0)	0.71 (0.62, 0.82)	0.078
Non-Hispanic Black	1703 (11.6)	1852 (11.9)	0.92 (0.82, 1.05)	0.0008
Non-Hispanic Asian	1062 (6.2)	863 (4.4)	0.65 (0.56, 0.74)	<0.0001
Other race ^3^	289 (4)	307 (3.5)	0.79 (0.62, 1.01)	0.93
Education level				
Less than high school	1904 (17.0)	1019 (9.6)	ref	
High school/GED	1807 (24.9)	1557 (20.7)	1.46 (1.28, 1.67)	0.040
Some college	2225 (30.8)	2582 (34.3)	1.96 (1.71, 2.26)	<0.0001
College graduate or above	1696 (27.3)	2204 (35.4)	2.29 (1.90, 2.75)	<0.0001
Poverty income ratio				
≥1.3 PIR	4155 (65.4)	5042 (77.6)	ref	
<1.3 PIR	3482 (34.6)	2320 (22.4)	0.55 (0.48, 0.61)	<0.0001
Marital status				
Married, living with partner	4782 (65.5)	4134 (61.0)	ref	
Not married	2849 (34.5)	3226 (39.0)	1.21 (1.09, 1.35)	0.0005
Country of birth				
Born in the US	4692 (76.8)	5591 (85.9)	ref	
Others	2939 (23.2)	1767 (14.1)	0.54 (0.48, 0.61)	<0.0001

^1^ ORs of >2 meals/week estimated using logistic regression models; ≤2 meals/week was used as reference. ^2^ Rao–Scott chi-square or logistic regression models. ^3^ Including multi-racial. FAFH: food away from home, prepared outside the home; LS: least squares; SE: standard error; OR: odds ratio; CI: confidential interval; GED: general educational development (equivalent to a high school degree).

**Table 2:** Differences in HEI-2015 scores by FAFH frequency.

	Least Squares Mean (Standard Error) ^1^		*p* ^1^
	FAFH ≤2 Meals/Week	FAFH >2 Meals/Week	
Total HEI-2015 score (MAX: 100)	52.1 (0.3)	49.5 (0.28)	<0.0001
Adequacy:			
Total Vegetables (MAX: 5)	3.06 (0.03)	2.98 (0.03)	0.031
Greens and Beans (MAX: 5)	1.84 (0.04)	1.64 (0.04)	<0.0001
Total Fruits (MAX: 5)	2.19 (0.04)	1.88 (0.05)	<0.0001
Whole Fruits (MAX: 5)	2.18 (0.04)	1.87 (0.05)	<0.0001
Whole Grains (MAX: 10)	2.52 (0.07)	2.00 (0.06)	<0.0001
Dairy (MAX: 10)	4.48 (0.07)	4.32 (0.06)	0.033
Total Protein Foods (MAX: 5)	4.24 (0.02)	4.28 (0.02)	0.224
Seafood and Plant Proteins (MAX: 5)	2.47 (0.05)	2.43 (0.04)	0.389
Fatty Acids (MAX: 10)	5.44 (0.07)	5.35 (0.06)	0.276
Moderation:			
Sodium (MAX: 10)	4.53 (0.06)	4.36 (0.06)	0.021
Refined Grains (MAX: 10)	5.74 (0.07)	5.56 (0.09)	0.067
Saturated Fats (MAX: 10)	6.54 (0.06)	6.25 (0.07)	0.0004
Added Sugars (MAX: 10)	6.86 (0.06)	6.57 (0.07)	0.0009

^1^ Regression models, adjusting for age, poverty income ratio, sex, race/ethnicity, education level, marital status, and country of birth. Underlined values mean rejecting null hypothesis after Bonferroni-Holm correction for multiple comparisons. HEI-2015: Healthy Eating Index-2015; FAFH: food away from home, prepared outside the home.

**Table 3:** Differences in anthropometric measures and biomarkers of chronic disease by FAFH frequency.

	Least Squares Mean (Standard Error) ^1^		*p* ^1^
	FAFH ≤2 Meals/Week	FAFH >2 Meals/Week	
Anthropometric measures			
BMI (kg/m^2^)			
Males	28.5 (0.18)	28.8 (0.18)	0.186
Females	29.4 (0.17)	30.0 (0.21)	0.030
Waist circumference (cm)			
Males	99.3 (0.47)	99.9 (0.42)	0.279
Females	96.2 (0.38)	97.5 (0.46)	0.044
Biomarkers			
Glycohemoglobin (%)	5.7 (0.03)	5.8 (0.03)	0.026
Total cholesterol (mg/dL)	191 (1.1)	193 (1.31)	0.141
Direct HDL cholesterol (mg/dL)	53 (0.38)	52 (0.47)	0.279
LDL cholesterol (mg/dL)	115 (0.98)	116 (1.07)	0.381
Triglyceride (mg/dL)	119 (2.88)	127 (3.19)	0.014

^1^ Regression models, adjusting for age, poverty income ratio, sex, race/ethnicity, education level, marital status, and country of birth. Underlined values mean rejecting null hypothesis after Bonferroni–Holm correction for multiple comparisons. FAFH: food away from home, prepared outside the home; BMI: body mass index; HDL: high-density lipoprotein; LDL: low-density lipoprotein.

## Data Availability

https://wwwn.cdc.gov/nchs/nhanes/continuousnhanes/default.aspx?BeginYear=2011, https://wwwn.cdc.gov/nchs/nhanes/continuousnhanes/default.aspx?BeginYear=2013, https://wwwn.cdc.gov/nchs/nhanes/continuousnhanes/default.aspx?BeginYear=2015, and https://wwwn.cdc.gov/nchs/nhanes/continuousnhanes/default.aspx?BeginYear=2017 (accessed on 29 November 2021).
